# Retroperitoneal bronchogenic cyst: a case report and literature review

**DOI:** 10.3389/fonc.2024.1406270

**Published:** 2024-10-31

**Authors:** Bohao Jiang, Tiantian Xie, Jiyuan Hu, Yitong Xu, Hao Zhang

**Affiliations:** ^1^ Department of Urology, The First Hospital of China Medical University, Shenyang, Liaoning, China; ^2^ Department of Anesthesiology, The First Hospital of China Medical University, Shenyang, Liaoning, China; ^3^ Department of Pathology, The First Hospital of China Medical University, Shenyang, Liaoning, China

**Keywords:** bronchogenic cyst, case report, literature review, retroperitoneal neoplasm, robot-assist surgery

## Abstract

**Introduction:**

Retroperitoneal bronchogenic cyst, typically situated in the subdiaphragmatic region, is a rare congenital benign developmental abnormality arising from dysplasia of the foregut and abnormal budding of the tracheobronchial tree. Due to its low incidence, there are limited reports regarding this condition.

**Case presentation:**

Four retroperitoneal bronchogenic cysts near the left adrenal gland were identified without accompanying clinical symptoms. One case was misdiagnosed as an adrenal tumor prior to surgery, while the others were diagnosed as retroperitoneal cysts of uncertain origin. All cases underwent surgical resection, with three being performed laparoscopically and one utilizing robot-assisted techniques. Pathological reports confirmed the diagnosis of bronchogenic cyst in each instance. The prognosis was favorable for all four patients, with no complications or recurrences observed. Additionally, a literature review was conducted, encompassing 82 cases, which revealed similar characteristics and radiological manifestations in the majority of cases.

**Conclusion:**

Although retroperitoneal bronchogenic cysts are rare developmental malformations lacking distinctive clinical and radiological features, reported cases exhibit similarities in certain clinical and imaging characteristics. This report offers additional insights into the diagnosis and management of this rare disease. Future reports are essential to enhance understanding of this disease.

## 1 Introduction

A bronchogenic cyst is a benign disease originating from the congenital dysplasia of the foregut in the embryonic stage. The location of the lesion is determined by the site of abnormal budding of the primitive tracheobronchial tube, which primarily occurs in the mediastinum and lungs (1). It has been reported that retroperitoneal bronchogenic cysts can also occur in other locations, including the neck, skin, spinal canal, and abdominal cavity (2–4). Retroperitoneal bronchogenic cysts, typically situated in the subdiaphragmatic region, often present as asymptomatic due to their slow growth rate and non-invasive nature. A small percentage of patients may experience symptoms such as pain, fever, and abdominal discomfort, which can result from compression of surrounding tissues or infection. Non-invasive diagnosis poses challenges due to the low incidence, subtle symptoms, and atypical radiological manifestations associated with these cysts. Surgical removal remains the primary treatment option for bronchogenic cysts to alleviate symptoms and establish a definitive diagnosis, despite the benign nature of the disease (5, 6). In recent years, laparoscopic and robot-assisted surgical techniques have become increasingly employed for the management of retroperitoneal bronchogenic cysts, owing to their reduced surgical trauma and improved recovery.

As discussed in the present report, four cases of retroperitoneal bronchogenic cysts were identified, all successfully surgically incised (3 cases under laparoscope and 1 case with the assistance of Da Vinci Xi robotic surgery system). We listed the basic characteristics and surgical information are summarized in **Table 1**. As a common method for eliminating the presence of functional adenomas or malignancies, laboratory results of adrenal function series and tumor biomarkers were also summarized. (**Supplementary Table S1**). Subsequently, a literature review was conducted to summarize the symptomatic features, imaging manifestations, surgical procedures, and duration of hospitalization reported in existing cases of this disease.

**Table 1:** Basic characteristics and surgical information of reported cases.

Case ID	Case 1	Case 2	Case 3	Case 4
Gender	F	F	M	F
Age	51	32	61	43
Side	L	L	L	L
Symptom	Asymptomatic	Asymptomatic	Asymptomatic	Asymptomatic
Hypertension	No	No	1 Year	No
Diabetes	No	No	1 Year	No
CHD	No	No	No	No
size (mm)	59.5*33.5	42*19*43	27*22*38	25*23*20
Routine laboratory test	N	N	N	N
adrenal gland function series	N	/	N	N
Tumor Biomarkers	/	N	/	N
Operation Type	Retroperitoneal Laparoscopy	Robot-assisted Laparoscopy	Retroperitoneal Laparoscopy	Retroperitoneal Laparoscopy
Surgery time (min)	65	84	180	64
Drainge volume (ml)	165	36	176	110
postoperation hospital stay	5 days	2 days	7 days	4 days
Total admission	12 days	6 days	11 days	13 days
Total cost($)	2698.7	7776.6	5997.3	4414.4

(CHD, Coronary Heart Disease).

## 2 Case 1

A 41-year-old female incidentally discovered a cystic tumor in the left adrenal area on a CT scan during a regular checkup offered by her company. She reported no positive symptoms, and a routine physical examination conducted upon hospitalization revealed no abnormal abdominal signs (**Table 1**). An irregular, slightly high-density shadow was detected in the left adrenal area (5.95*3.35cm, HU=64) on enhanced CT with multiple calcifications. No significant enhancement was observed. Retroperitoneal lymph nodes were not detected as enlarged. Given the potential for adrenal-derived tumors, a series of adrenal gland hormone tests was conducted. Angiotensin I (clinostatism: 0.89ng/ml) and angiotensin II (standing position: 44ng/ml; clinostatism: 57ng/ml) were slightly elevated.

**Figure 1:**
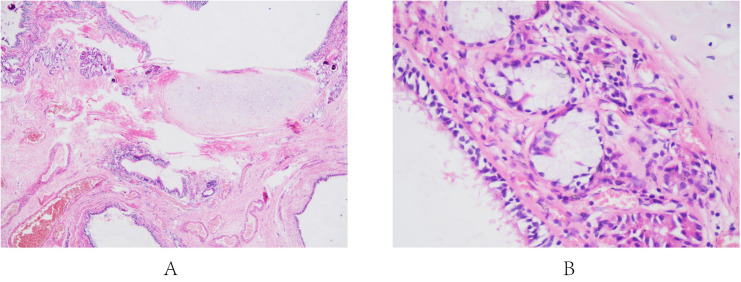
Pathological section images of Case 1. (**A**: low-power magnification [40×]: Cyst cavity covered with ciliated columnar epithelium. **B**: high-power magnification [100×]: Bronchial glands and cartilage tissue with the dilation and congestion of surrounding blood vessels).

In light of the preliminary diagnosis of an adrenal tumor, a retroperitoneal laparoscopic left adrenalectomy was performed with the patient in a right-side lying position (**Table 1**). Following an incision of the renal fascia, a cyst measuring approximately 5.0*3.5 cm was identified near the middle part and lateral branch of the adrenal gland. After dissecting the central vein of the left adrenal gland and securing it with two hem-locks at the proximal end, both the left adrenal gland and the tumor were excised together. The cyst wall was smooth, containing a light brown viscous fluid. Under the microscope, the structures of the cyst cavity were observed, covered with ciliated columnar epithelium (**Figure 1A**). Within the fibrous cyst wall tissue, bronchial glands and cartilage tissue were presented with the dilation and congestion of surrounding blood vessels (**Figure 1B**). The characteristic bronchial tissue structure confirmed the diagnosis of a bronchogenic cyst. No adverse events were observed post-surgery, and the one-year follow-up examination after discharge indicated no recurrence.

## 3 Case 2

A 32-year-old female patient presented with a retroperitoneal mass, initially suspected to be a benign adrenal tumor. The mass was incidentally detected during an ultrasound examination. The patient denied a history of medication use or chronic disease. Physical examination and laboratory tests revealed no significant abnormalities (**Table 1**). Serum tumor markers were tested to rule out the possibility of a malignant tumor, and no positive results were obtained.

**Figure 2:**
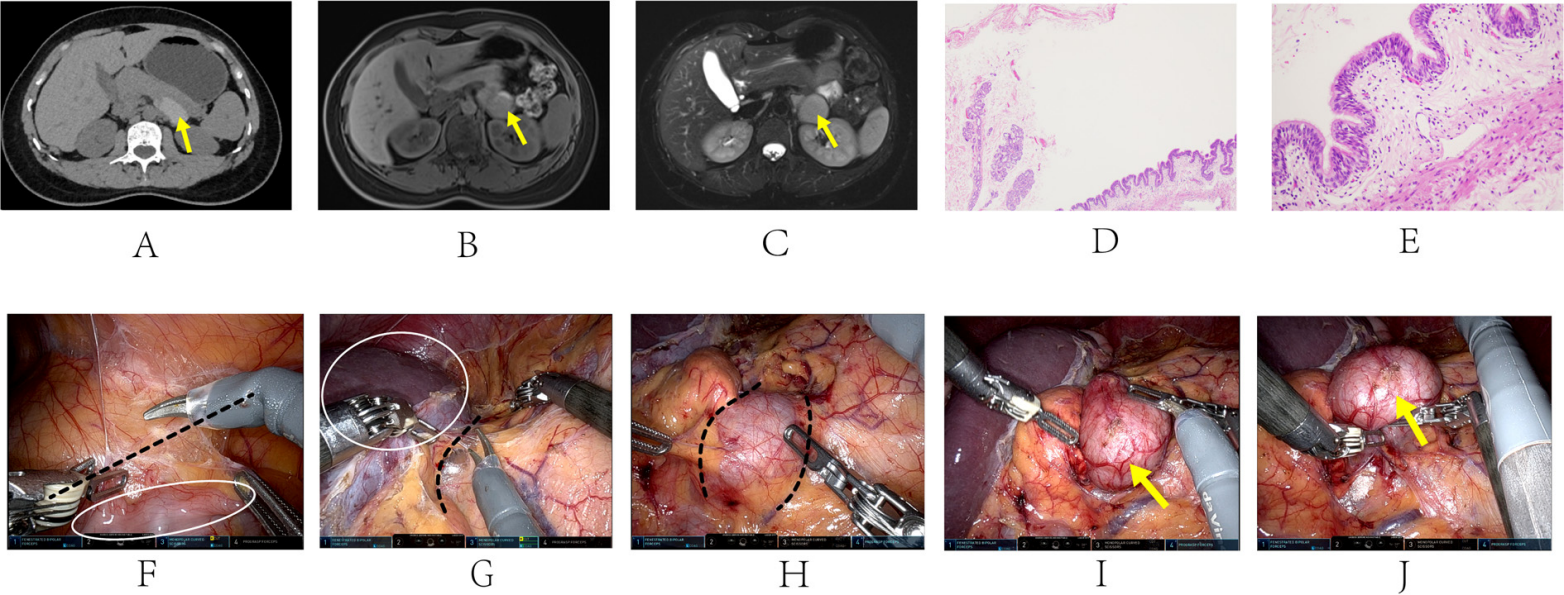
Radiological and pathological manifestations of Case 2 **(A-E)**. Procedure of the robot-assisted surgery **(F-J)**. The yellow arrow shows the location of the cyst: **A**: CT scan showed an ovoid, high-dense 4.2*1.9*4.3cm mass adjacent to abdominal aorta, with a HU value of 90; **B**: MRI image showed a 3.7*3.3*4.2cm cyst with a short signal at the T1-weighted stage; **C**: The cyst presented as a long signal on the MRI image at the T2-weighted stage, indicating the presence of mucinous cystic content. **D**: low-power magnification [40×]: Large cystic structures covered with characteristic ciliated columnar epithelium. **E**: high-power magnification [100×]: Mucous glands surrounded by smooth muscle tissue and cystic wall tissue. Fascia and soft tissue were separated along the black dashed line in image **(F-H)**. **(F)** lift the lateral mesocolon of the descending colon, incise the left paracolic groove open. White circle: colon; **(G)** separate the anterior layer of the perirenal fascia and the plane of digestive system. White circle: spleen; **(H, I)** separate the tumor from the tail of the pancreas and the left perirenal fascia; **(J)** excise the cyst.

To further clarify the diagnosis, the patient received CT and MRI examination (**Figure 2**). During CT examination, an ovoid, high-density cystic lesion measuring 4.2*1.9*4.3cm, with a HU value of 90, was detected in the left adrenal area adjacent to the abdominal aorta. Focal calcifications were observed in the wall of the cyst. Enhanced MRI imaging revealed a well-defined cyst that showed no enhancement. The lesion appeared as a short signal on T1-weighted images and a long signal on T2-weighted images, indicating the presence of mucinous cystic content in the lesion.

A robot-assisted laparoscopic retroperitoneal mass resection was performed (**Table 1**). The surgery was performed with the patient in a supine position with the operating table tilted 70° to the right. Five incisions were selected. The selection of incision locations is shown in **Supplementary Figure S1**. A 0.6cm incision was made separately at points A, B, C, and D, and an 8mm Trocar was placed at each point. A 1.0cm skin incision was made at point E, and a 12mm Trocar was placed. After docking the robotic arm system at the side of operating table, a laparoscopic lens was placed at point C, and other surgical instruments such as scissors, bipolar forceps, separation forceps, and suction rods were placed at other points.

The procedure of the surgery is presented (**Figure 2** and **Supplementary Video S1**). The lateral mesocolon of the descending colon was elevated, and the left paracolic groove was incised, allowing for the separation of the anterior layer of the perirenal fascia. The spleen and pancreas were retracted posteriorly, revealing a cystic tumor located between the tail of the pancreas and the left perirenal fascia. The tumor exhibited spontaneous undulation under the laparoscope. Complete excision of the tumor was successfully performed, and it was placed in a specimen bag for removal through an extended incision at point D. A drainage tube was positioned at point B, and the five incisions were sutured layer by layer. Gross examination of the excised cystic tumor revealed dimensions of approximately 5.5*5.0*3.5cm. Upon incision of the specimen, yellow cystic fluid was noted. Under the microscope, large cystic structures were observed, covered with characteristic ciliated columnar epithelium. Mucous glands could be seen below, surrounded by smooth muscle tissue and cystic wall tissue, confirming the diagnosis of a retroperitoneal bronchogenic cyst (**Figures 2D, E**). The patient was discharged without complications on the second day following the surgery.

## 4 Case 3

**Figure 3:**
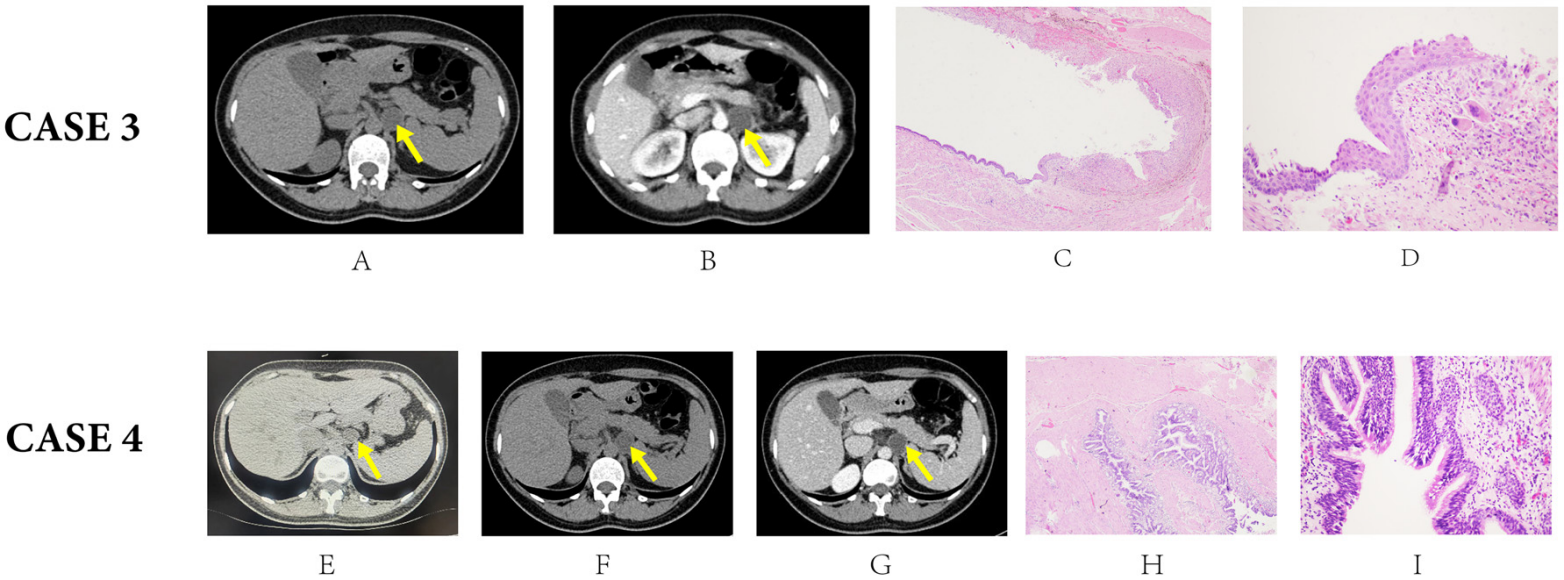
CT and pathological manifestations of Case 3 **(A-D)** and Case 4 **(E-I)**. The yellow arrow shows the location of the cyst: (Case 3: **(A)** non-contrast enhanced stage: A 31HU 2.7*2.2cm soft-tissue nodule in the left adrenal area; **(B)** contrast enhanced stage: insignificant enhancement after contrast agent injection; **(C)**: low-power magnification [40×]: The capsule wall was surrounded with smooth muscle and fibrous connective tissue. **(D)**: high-power magnification [100×]: Migration area of ciliated columnar epithelium, squamous epithelium, and foam cell surrounded by deposition of hemosiderin and vasodilation congestion. Case 4: Image **(E)** was taken two years ago when the mass was first detected. Image **(F, G)** were taken in our outpatient clinic at the time of admission. The yellow arrow shows the location of the cyst: **(E)** The CT image showed a 2.0*2.1 cm ovoid low-density mass. **(F)** non-contrast enhanced stage: A low-density mass in the left adrenal area with a diameter of 2.5 cm; **(G)** contrast enhanced stage: Insignificant enhancement after contrast agent injection. H: low-power magnification [40×]: Cystic structure was surrounded by smooth muscle with ciliated columnar epithelium inside the cyst wall. I: high-power magnification [100×]: Infiltration of mucinous glands and lymphocytes).

A 61-year-old male patient presented at the outpatient clinic with a complaint of a retroperitoneal mass, which was incidentally discovered on a CT scan performed two weeks prior for an abdominal impact injury. The patient exhibited no apparent symptoms at the time of admission, and no abnormal physical signs were observed in the abdominal area (**Table 1**). Laboratory tests and a series of adrenal gland hormone tests were conducted, and negative results were reported. Preliminary diagnosis suggested a non-functional retroperitoneal tumor originating from the left adrenal gland or pancreas. An enhanced CT scan was performed to further confirm the diagnosis (**Figures 3A, B**). The scan revealed an oval nodule in the retroperitoneum, measuring 2.7*2.2cm, with a HU value of 31, and showed minimal enhancement after intravenous administration of the contrast agent, yielding a HU value of 37.

To exclude the possibility of non-functional adrenal adenoma, the patient underwent laparoscopic retroperitoneal tumor resection in a right lateral position (**Table 1**). The perirenal fascia was incised to expose the left adrenal area, revealing a retroperitoneal tumor with a smooth capsule located in the triangular space formed by the renal artery, vein, and adrenal gland. The tumor measured approximately 3.5*3.0cm. After carefully dissecting the left renal artery, left renal vein, and adrenal gland, the tumor was removed entirely. Microscopically, a cystic mass was seen with smooth muscle and fibrous connective tissue surrounded by the capsule wall (**Figure 3C**). The capsule cavity was composed of a large number of ciliated columnar epithelium. The stratified squamous epithelium was also seen. A large area of foam cell deposition was presented, surrounded by deposition of hemosiderin and vasodilation congestion. Under immunohistochemical staining, foam cells were CD68 positive expression, supporting the histocyte phenotype. At high-power magnification, **Figure 3D** showed the migration area of ciliated columnar epithelium, squamous epithelium, and foam cell, confirming the diagnosis of a bronchogenic cyst. Regular follow-up after discharge showed no complications and no other apparent abnormalities.

## 5 Case 4

A 43-year-old female patient presented to the hospital with a 2-year history of a retroperitoneal mass, which was identified on a CT scan during a routine physical examination. Compared to the CT image when the mass was first detected (**Figure 3E**), the mass had increased in size over the past two years. The patient reported no positive symptoms and had no history of chronic diseases. Laboratory tests conducted upon admission yielded normal results (**Table 1**). To exclude the possibility of a functional or malignant adrenal tumor, a series of adrenal gland hormone tests and tumor biomarkers were performed, all of which returned negative results. CT showed a low-density cystic mass in the left adrenal area with a diameter of 2.5 cm without significant enhancement during the enhancement phase. The border with the left adrenal gland was unclear (**Figures 3F, G**).

Considering the continuous enlargement of the mass, a laparoscopic retroperitoneal mass resection was performed on a right-side lying position (**Table 1**). A cystic tumor was visible in front of the psoas major muscle, measuring approximately 2.0*2.0cm, which had a clear border with the left adrenal gland. After opening the excised tumor, the cyst wall was smooth and contained yellow cystic fluid. Under the microscope, the cystic structure was surrounded by smooth muscle with ciliated columnar epithelium inside the cyst wall, accompanied by infiltration of mucinous glands and lymphocytes, confirming the diagnosis of bronchogenic cysts. (**Figures 3H, I**). The patient was discharged 4 days after surgery with a good prognosis. Regular follow-up was accomplished through post-operative telephone surveys, the results of which remain uneventful until now. The patient was also recommended for a post-operative assessment at the outpatient of her local hospital 6 months after surgery, and no recurrence was indicated.

## 6 Discussion

A bronchogenic cyst is a benign cystic mass originating from abnormal budding and isolation of bronchial buds in the fifth week of embryonic formation (7). Typically, the derivatives of isolated buds are located near the trachea and bronchus in the mediastinum, where the majority of bronchogenic cysts are found. However, in rare cases, the connection to the primitive tracheobronchial tree may be severed, allowing these cysts to migrate to other anatomical locations, such as the neck, skin, and abdominal cavity, as documented in the literature (2–4, 8). Retroperitoneal bronchogenic cyst is a rare subtype caused by the fusion of pleuroperitoneal membranes, which usually happens at the end of the 6th week of embryonic development. The pericardial-peritoneal canal is divided into two independent cavities by the fusion of the pleuroperitoneal membrane, a process that plays a crucial role in diaphragm formation. During this process, the primitive respiratory trees are pinched off by these membranes and become positioned within the retroperitoneal space, which gradually develops into retroperitoneal bronchogenic cysts as the embryo matures (7).

Several literature reviews have been conducted to summarize the characteristics of retroperitoneal bronchogenic cysts (8–12). However, previous literature reviews exhibit limitations, such as focusing exclusively on cases with specific features or surgical methods and often omitting cases reported after their reviews were published. To provide a more comprehensive summary, a literature review of retroperitoneal bronchogenic cysts was conducted, encompassing all reported cases. This review cataloged their respective publication years, chief complaints, surgical procedures, laboratory test results, and other relevant characteristics. The findings of this article significantly complement earlier literature reviews and offer additional insights into the therapeutic process.

Thesaurus terms “Bronchogenic Cyst” on the MeSH database and 3 free words “Bronchogenic Cysts, Bronchial Cyst, and Bronchial Cysts” were applied for literature search on the PubMed website, with the location of mass restricted to retroperitoneum. The search formula is “((((Bronchogenic Cyst) OR (Bronchogenic Cysts)) OR (Bronchial Cyst)) OR (Bronchial Cysts)) AND (retroperitoneal)”. Due to the rarity of retroperitoneal bronchogenic cysts, only 92 case reports have been published in English, totaling 101 cases as indicated by research results from the PubMed database covering the period from 1991 to 2024. After screening the full texts, restricting the mass location to the retroperitoneal area, and excluding reports with incomplete pathological information, 74 publications were included in this literature review, which collectively reported 82 cases of retroperitoneal bronchogenic cysts. Detailed information and summarized characteristics of the included articles are shown in the **Supplementary Table S2**.

From a demographic perspective, retroperitoneal bronchogenic cysts do not appear to be associated with susceptibility in any specific population. As shown in **Supplementary Figure S2**, there have been reports of confirmed retroperitoneal bronchogenic cysts of all ages, from the prenatal stage to 75 years old, with an average diagnosed age of 40.2. Most retroperitoneal bronchogenic cysts were identified in middle-aged individuals, with 29 cases (35%) occurring in the 21-40 age group and 35 cases (43%) in the 41-60 age group. This prevalence may be attributed to the continuous enlargement of the cysts and the increasing incidence of other related diseases. Regarding incidence rates, there was no significant difference between genders, with 41 cases reported in females and 41 in males. Additionally, these cysts were more commonly located on the left side (72 cases, 88%) compared to the right side (9 cases, 11%) and the midline (1 case, 1%), which aligns with our reports (**Table 1**) and findings from previous literature reviews (13, 14).

The symptoms of retroperitoneal bronchogenic cysts are typically non-specific. Nearly half of the cases were discovered incidentally during routine physical examinations (43 out of 82 cases, or 53%). The most common chief complaint associated with this condition was abdominal pain, reported in 28 cases (34%), followed by abdominal or thoracic discomfort, noted in 6 cases (7%). A very small number of cases were identified during medical evaluations prompted by other symptoms, which may arise from infections or oversized cysts. These symptoms included fever (15), recurrent urinary tract infection (9), dyspeptic symptoms (16) and limb numbness (17). Notably, the chief complaint of pain was not always caused by the cyst itself but rather by other underlying diseases. For example, Joshua et al. reported a case involving complaints of pain during defecation, constipation, and bloody stools, which were ultimately diagnosed as right colon adenocarcinoma upon further examination (18).

Making a presurgical radiological diagnosis is challenging due to the atypical imaging features of retroperitoneal bronchogenic cysts. On CT images, the mass typically presents as a rounded or ovoid low-density cyst that exhibits minimal to no enhancement (19). Significantly, in some cases with high-level protein content in the cyst fluid, soft-tissue density shadows can be found in the images (8). Similar to the CT manifestations, MRI images of retroperitoneal bronchogenic cysts also lack distinctive diagnostic features. They typically display high signal intensity on T1-weighted images and low to intermediate signal intensity on T2-weighted images, with no enhancement observed following the injection of gadolinium (20). Similar radiological manifestations were also observed in the four present cases.

Due to the low incidence rate, uncharacteristic symptoms, and atypical imaging features, this disease is often misdiagnosed as cysts and solid tumors originating from nearby organs, such as the adrenal gland and pancreas. Surgical resection and subsequent pathologic examination remain the only definitive methods for confirming the diagnosis of retroperitoneal bronchogenic cysts. Microscopic analysis typically reveals epithelial tissue, smooth muscle of the respiratory tract, cartilage, and respiratory glands within the cyst (8). Although retroperitoneal bronchogenic cysts are classified as benign with an extremely low risk of malignant transformation, surgical removal is strongly recommended to confirm the diagnosis, alleviate symptoms, and prevent potential complications and malignant transformation. Resection of these cysts can be performed using either laparotomy or laparoscopy techniques. According to the present literature review results (**Supplementary Figure S2**), 55% of the population has undergone laparoscopic surgery for treatment (45/82, 55%), which is widely believed to help reduce intraoperative bleeding and improve prognosis. With the development of surgical instruments and the maturation of laparoscopic surgery techniques, the proportion has increased to 68% in the past 20 years. In addition, robot-assisted surgeries have been extensively adopted in recent years. Three cases of robotic resection were previously reported; however, they were not included in the present literature review due to the absence of pathological images provided (21, 22). The present report (Case 2) documented an excellent post-operative outcome following robot-assisted surgery, characterized by low drainage volume and a short postoperative hospitalization period. Additionally, no significant differences were observed in the duration of surgery or total hospitalization costs compared to laparoscopic surgery. Almost all previous reports indicate that the prognosis for retroperitoneal bronchogenic cysts is generally favorable, with no occurrences of malignancy or recurrence. However, Sullivan et al. reported a case involving a 55-year-old patient who experienced malignant transformation into adenocarcinoma, underscoring the importance of surgical resection and regular postoperative follow-up (23). To date, there is no clear consensus on the optimal post-operative follow-up frequency for this disease. As the possibility of recurrence and malignancy after complete removal of the cyst is extremely low, we recommend one postoperative radiological checkup six months after surgery. The frequency of subsequent follow-ups may be reduced to once a year in the absence of evidence of recurrence.

The present case report has several advantages. First, while there have been previous reports of robotic surgery for retroperitoneal bronchogenic cysts, the present report is the first to provide a detailed account of the entire surgical procedure using the Da Vinci Xi robotic system. Compared with two previous reports, original pathologic images and a surgical video are also provided (21, 22). Second, in addition to reporting routine postoperative data (admission time and prognosis), detailed postoperative data that were rarely mentioned in other articles were also reported. The data included surgical time, volume of drainage, and total cost of hospitalization, which may provide information on the selection of protocols for laparoscopic or robotic surgery for this disease. Third, a literature review was conducted in which all case reports to date in the Pubmed database were included. Compared with previous reviews, more cases were included, and laboratory results of adrenal function series and tumor biomarkers were collected for each case, providing a more comprehensive summary of the disease’s characteristics. However, the present report has certain limitations. Although promising surgical outcomes were observed in the case that underwent robotic resection, including shorter postoperative hospitalization and faster recovery compared to laparoscopic surgeries, the findings are not sufficiently convincing due to the limited number of reported cases involving robotic surgery. Future case reports on retroperitoneal bronchogenic cysts, especially those focusing on laparoscopic and robotic-assisted techniques, are urgently needed to enhance the understanding and treatment of this condition.

In conclusion, retroperitoneal bronchogenic cysts are rare congenital benign conditions that lack characteristic symptoms and typical radiological manifestations. Surgical resection of these cysts is recommended to confirm the pathological diagnosis, alleviate symptoms caused by compression, and prevent potential malignant transformation despite the generally favorable prognosis following surgery. There is a pressing need for future reports on retroperitoneal bronchogenic cysts to enhance the understanding of their characteristics and diagnostic criteria.

## Data Availability

The original contributions presented in the study are included in the article/**Supplementary Material**. Further inquiries can be directed to the corresponding authors.
