# Supplementary figures and images for: Retroperitoneal bronchogenic cyst: a case report and literature review
Source: Front Oncol. 2024 Oct 31;14:1406270. doi: 10.3389/fonc.2024.1406270 (PMC11560907; doi:10.3389/fonc.2024.1406270)

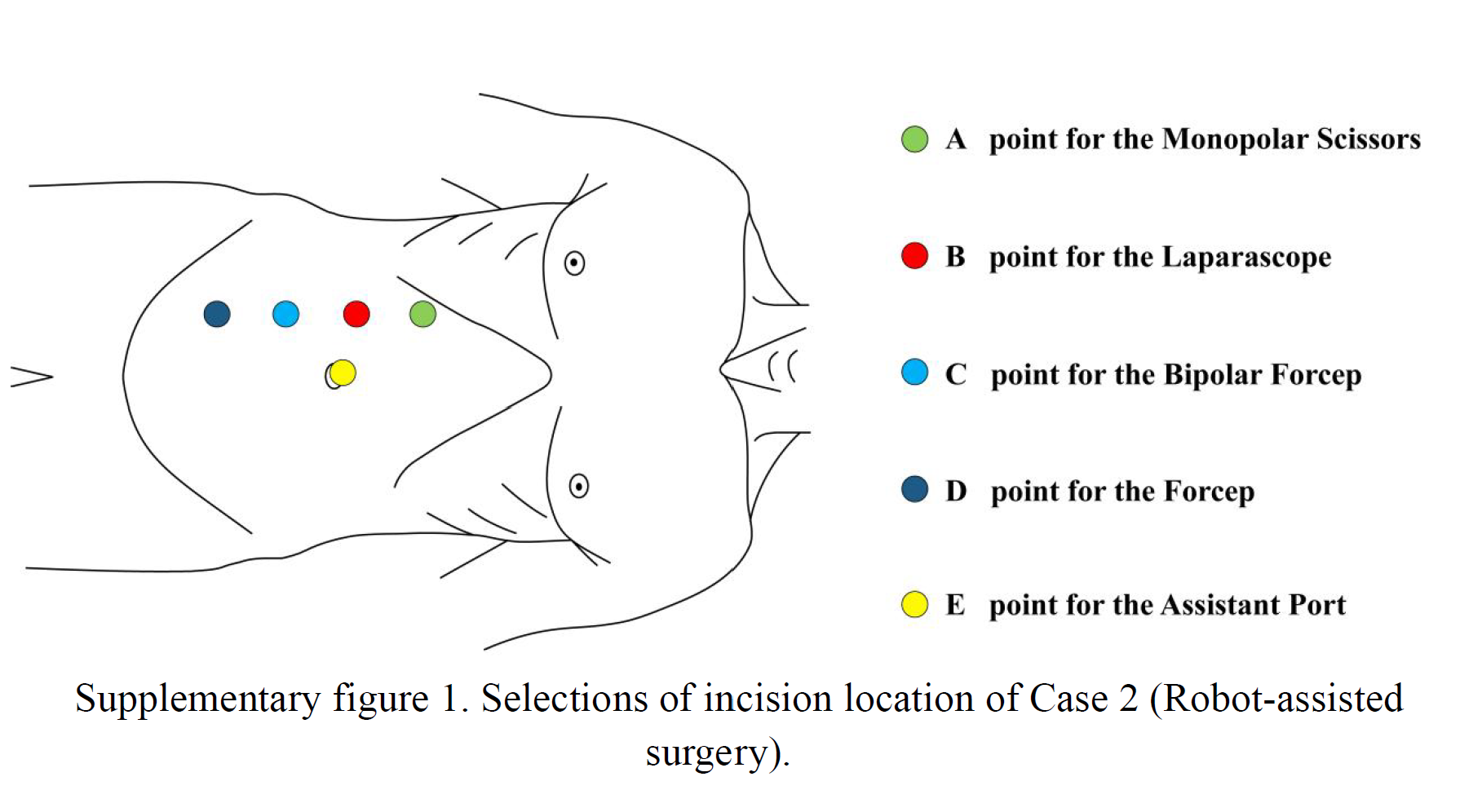

Supplement: Supplementary file 1 [file Image1.png]

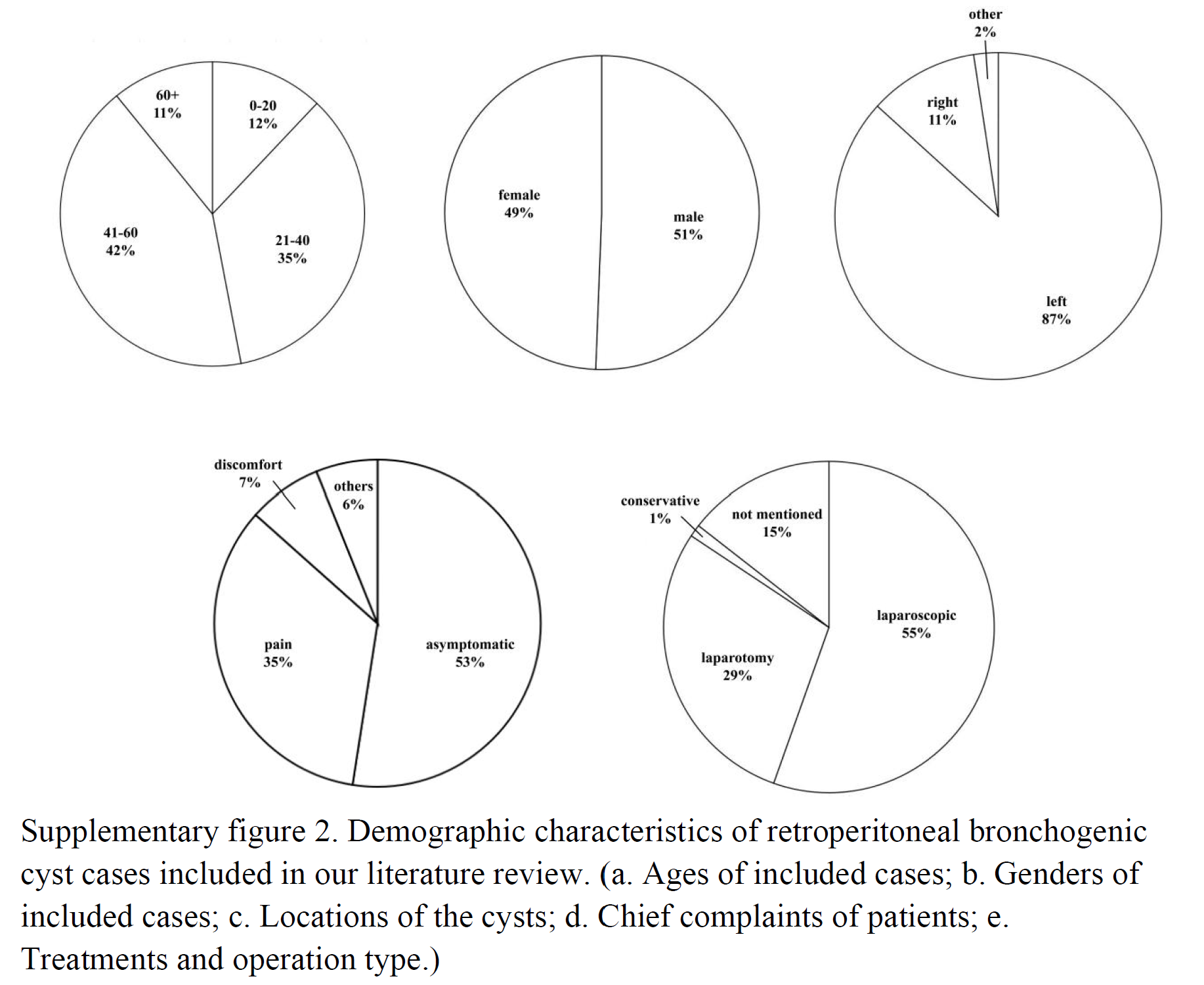

Supplement: Supplementary file 2 [file Image2.png]
